# Anticancer property of *Bryophyllum pinnata *(Lam.) Oken. leaf on human cervical cancer cells

**DOI:** 10.1186/1472-6882-12-15

**Published:** 2012-03-10

**Authors:** Sutapa Mahata, Saurabh Maru, Shirish Shukla, Arvind Pandey, G Mugesh, Bhudev C Das, Alok C Bharti

**Affiliations:** 1 Division of Molecular Oncology, Institute of Cytology and Preventive Oncology (ICMR), I-7, Sector-39, Noida 201301, INDIA; 2 Department of Inorganic and Physical Chemistry, Indian Institute of Science, Bangalore 560 012, INDIA; 3 Dr. B.R Ambedkar Centre for Biomedical Research, University of Delhi (North Campus), Delhi 110007, INDIA

**Keywords:** *Bryophyllum pinnata*, Human Papillomavirus, HeLa cells, AP1, NMR, HPTLC

## Abstract

**Background:**

*Bryophyllum pinnata *(*B. pinnata*) is a common medicinal plant used in traditional medicine of India and of other countries for curing various infections, bowel diseases, healing wounds and other ailments. However, its anticancer properties are poorly defined. In view of broad spectrum therapeutic potential of *B. pinnata *we designed a study to examine anti-cancer and anti-Human Papillomavirus (HPV) activities in its leaf extracts and tried to isolate its active principle.

**Methods:**

A chloroform extract derived from a bulk of botanically well-characterized pulverized *B*. *pinnata *leaves was separated using column chromatography with step- gradient of petroleum ether and ethyl acetate. Fractions were characterized for phyto-chemical compounds by TLC, HPTLC and NMR and Biological activity of the fractions were examined by MTT-based cell viability assay, Electrophoretic Mobility Shift Assay, Northern blotting and assay of apoptosis related proteins by immunoblotting in human cervical cancer cells.

**Results:**

Results showed presence of growth inhibitory activity in the crude leaf extracts with IC_50 _at 552 μg/ml which resolved to fraction F4 (Petroleum Ether: Ethyl Acetate:: 50:50) and showed IC_50 _at 91 μg/ml. Investigations of anti-viral activity of the extract and its fraction revealed a specific anti-HPV activity on cervical cancer cells as evidenced by downregulation of constitutively active AP1 specific DNA binding activity and suppression of oncogenic c-Fos and c-Jun expression which was accompanied by inhibition of HPV18 transcription. In addition to inhibiting growth, fraction F4 strongly induced apoptosis as evidenced by an increased expression of the pro-apoptotic protein Bax, suppression of the anti-apoptotic molecules Bcl-2, and activation of caspase-3 and cleavage of PARP-1. Phytochemical analysis of fraction F4 by HPTLC and NMR indicated presence of activity that resembled Bryophyllin A.

**Conclusions:**

Our study therefore demonstrates presence of anticancer and anti-HPV an activity in *B*. *pinnata *leaves that can be further exploited as a potential anticancer, anti-HPV therapeutic for treatment of HPV infection and cervical cancer.

## Background

For many centuries, plants have been a rich source of therapeutic agents and provided basis for several synthetic drugs. Despite great development of organic synthesis, currently 75% of prescribed drugs worldwide are derived from plant sources [1], showing that plant species are still an important source of new drugs for diseases that continue to lack a cure, such as cancer.

*Bryophyllum pinnatum *(Lam.) (synonym: *Kalanchoe pinnata*, Lam.; common names: Life plant, air plant (Mexican), love plant, Canterbury bells, Cathedral bells, etc) is a perennial herb that grow in the wild and used as a traditional medicinal plant in tropical Africa, China, Australia, tropical America and Indian system of medicine- Ayurveda. The leaf extracts of this plant have been routinely used for ailments like bacterial, fungal and viral infections, asthma, kidney stones, and ulcers. The leaves of this plant have been reported to possess antimicrobial [2], antifungal [3], anti-ulcer [4], anti-inflammatory and analgesic [5,6], antihypertensive [7], antidiabetic [8] and antimutagenic activities [9]. A number of active compounds, including flavonoids, glycosides, steroids, bufadienolides and organic acids have been identified in *B. pinnata *[10-12] that have been shown individually to possess variety of activities such as antibacterial, antitumor, cancer preventive and insecticidal actions. The flavonoid glycoside, Quercitrin (quercetin 3- O- alpha- L-rhamnopyranoside) and skap innato side were isolated with anti-leishmanial activity [13-17]. Despite having broad spectrum therapeutic potential, *B. pinnata*'s anticancer activity in general and anti-HPV activity in particular have not been explored as yet.

Cervical cancer is the principal cause of cancer-related mortality in women of the developing countries that contribute more than 85% of global disease burden [18]. Persistent infection with high-risk human papillomavirus (HR-HPV), most notably of the type 16 and 18, is an essential prerequisite for the development of cervical cancer [19-23] resulting in dysregulation of host cell cycle by targeting pivotal cell cycle proteins p53 & pRb by their viral gene products E6 & E7, respectively. Constitutive expression of HR-HPV E6 and E7 oncogene is mainly dependent on the availability of host cell transcription factors that act upon viral promoter and enhancer region. Activator protein-1 (AP1), a heterodimer of a group of structurally and functionally related members of the Jun (c-Jun, JunB, JunD) and Fos family (c-Fos, FosB, Fra-1 and Fra-2) is one of the transcription factors that are essentially required for viral oncogene expression [24]. Mutational inactivation of AP1 cis-acting site within the HR-HPV upstream regulatory region (URR) that facilitates AP1 interactions revealed a complete loss of transcriptional activity of the E6/E7 promoter and showed a key role of AP1 in HPV-mediated carcinogenesis [25]. Studies by our group demonstrated a significant overexpression of constitutively active AP1 family members in cervical precancer and cancer tissues [26]. Though HPV-mediated mechanism of cervical carcinogenesis is now well defined, anti-HPV therapeutics for elimination of HPV infections are yet to become a clinical reality and conventional physical ablation of HPV- infected lesions in precancer stage are in clinical practice [27]. Currently, there is no HPV specific advanced therapy for treatment of cervical cancer. Recent studies have shown that anti-oxidants and herbal derivatives may be effective against prevailing HR-HPV infection but these leads are yet to prove their efficacy in preclinical and subsequent clinical studies [26-29].

In view of absence of anti-HPV therapeutic for prevention and treatment of cervical cancer, in the present study, we examined leaves of *B. pinnata *for presence of anti-cancer and anti-HPV activity against human cervical cancer cells, HeLa that harbors HR-HPV type 18.

## Materials and methods

### Materials

The HPV18 positive human cervical cancer cell line, HeLa was obtained from the American Type Culture Collection (ATCC), USA. Polyclonal antibodies to Caspase-3, PARP-1, Bax, Bcl-2, β *-*actin, c-Jun, JunB, JunD, and c-Fos were purchased from Santa Cruz Biotechnology (Santa Cruz, CA). Dulbecco's modified Eagle's medium (DMEM), Fetal calf serum (FCS), 3-(4, 5- dimethylthiazol-2-yl) -2, 5-diphenyltetrazolium bromide (MTT), penicillin-streptomycin solution and all other reagents used in present study were of analytical grade and purchased from Sigma (St Louis, MO).

### Collection of plant material and identification

The leaves of *B. pinnata *were collected from Indore region of Madhya Pradesh (MP), India in the month of March-April, identified and authenticated by the Department of Botany, Devi Ahilya Vishwavidyalaya, Indore, Madhya Pradesh, India. The collected plant material was made free from foreign organic matter.

### Extraction and separation through column chromatography

**Figure 1:**
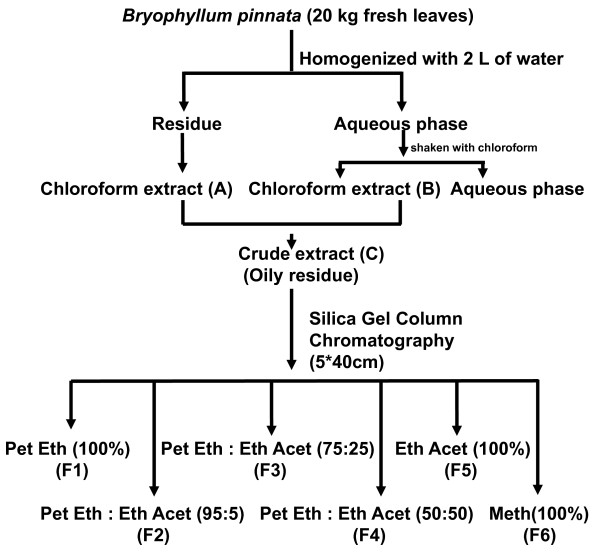
**Flow chart of extraction and column chromatographic separation of *Bryophyllum pinnata *leaves**. 20 kg fresh leaves of *B. pinnata *were crushed with water and filtered. Residue was dried and extracted with chloroform and fractionated with gradient of petroleum ether and ethyl acetated followed by washing with methanol. Fractions were designated as F1 to F6 as indicated in the figure. Pet Eth, Petroleum Ether; Eth Acet, Ethyl Acetate; Meth, Methanol.

Fresh leaves (20 kg) were crushed, washed with water and filtered. After filtration, the residual leaf material was dried and extracted with chloroform (Soxhlet extraction). The chloroform extract then dried and designated A. On the other hand, the filtered water was shaken with chloroform. The chloroform layer then separated from water layer, dried and designated B. Finally, both the extract A and B were mixed together and designated C (26.44 gm). The C was separated through silica gel column chromatography with the solvent petroleum ether (100-0%) to ethyl acetate (0-100%) in gradient step and final elution was performed with 100% methanol. Thus resulting in 6 different fractions that were designated from Fraction F1 to Fraction F6 (Figure 1). Finally fractions were concentrated under vacuum.

### Thin layer chromatographic (TLC) analysis

All the fractions were applied on to the precoated silica gel TLC plates and chromatographed using the solvent system chloroform: methanol (95:5). Plates were examined under UV and visible light as described earlier.

### Phytochemical analysis

Specimen from each fraction was examined to check the presence of bioactive phytochemicals using different chemical tests. Raymond test was performed for detecting the presence of steroidal glycosides. Briefly, 0.5 g of the extracts was dissolved in 1 ml of 50% ethanol and 0.1 ml 1% solution of metadinitrobenzene in methanol. 2-3 drops of 20% sodium hydroxide were added that lead to violet coloration which changed to blue with presence of steroidal glycosides. Dragendorff's test was performed for detecting the presence of alkaloids. Briefly, 0.5 g of dried extracts was dissolved in methanol and added with Dragendorff's reagent. Turbidity and precipitation indicated the positive test for alkaloids.

### Nuclear Magnetic Resonance (NMR) spectroscopy analysis

Specimens from each fraction were subjected to Proton NMR (Bruker AMX-400 NMR spectrometer). (^1^H NMR) spectra were recorded in CDCl3 on 400 MHz. Chemical shifts are cited in ppm with respect to SiMe4 as an internal control.

### High Performance Thin Layer Chromatographic (HPTLC) analysis

Silica gel 60 F_254 _(10 cm × 10 cm; 2 mm thickness, E. Merck, Darmstad, Germany) was used as stationary phase and extract band (application volume 8 μl, width 8 mm, space between bands 14 mm) was applied on the plate with CAMAG Micro-liter syringe. Chloroform: methanol (9.5:0.5 v/v) was used as mobile phase. Chamber was saturated for 15 min at room temperature. The length of chromatogram run was 85 mm. Plate was developed for 90 min in Twin trough glass chamber (CAMAG, Muttenz, Switzerland). Densitometric analysis was done with CAMAG TLC scanner III with scanning speed of 20 mm/sec at detection wave length of 360 nm and 615 nm by employing D2 & W lamp as radiation source and the procedure were operated by winCATS software (Ver. 1.2.0). Chromatogram recorded on two modes, first before and then after spraying anisaldehyde-sulphuric acid (AS) reagent. CAMAG Linomat-V application (CAMAG, Muttenz, Switzerland) was used for all above analysis.

### Cell culture

HeLa cells were cultured in DMEM supplemented with 10% FCS, 100 U/ml penicillin, 100 μg/ml streptomycin and maintained at 37°C in a humidified atmosphere of 5% CO_2 _as recommended by ATCC. *B. pinnata *was freshly dissolved in DMSO prior to cell culture treatment and cells were treated with variable concentration of *B. pinnata *crude extract and its fractions (F1-F6) and examined for viability, AP-1 DNA binding activity and other molecular assays as described.

### MTT assay

The cells (1 × 10^3^) seeded in 96-well plate and grown overnight, were treated with crude extract and different fractions at indicated concentration for 24 h, 48 h and 72 h. Two hours prior to completion of treatment duration, cultures were supplemented with MTT. After the incubation at 37°C, the cells were lysed with lysis buffer containing 50% of dimethyl formamide and 20% SDS and absorbance was measured at 570 nm using microtitre plate reader (Biotek, Winooski, Vermont) as described earlier [28]. The percentage of cell viability was calculated using the following formula: Percentage cell viability = (OD of the experiment samples/OD of the control) × 100. IC_50 _was calculated by linear interpolation method using the formula: IC_50 _= [(50-A)/(B- A)] × (D-C) + C, where A = the first point on the curve, expressed in percent inhibition, that is less than 50%; B = the first point on the curve, expressed in percent inhibition, that is greater than or equal to 50%; C = the concentration of inhibitor that gives A% inhibition; and D = the concentration of inhibitor that gives B% inhibition [30].

### Isolation of Nuclear Proteins and Electrophoretic mobility shift assay (EMSA)

Nuclear proteins were isolated from treated and control HeLa cells and were subjected to EMSA to assess AP1 -specific DNA binding as previously described [28]. Briefly, cells treated with different concentration of crude extract and fraction F4 for 24 hours were harvested and then nuclear extracts were prepared. The protein concentration of the extracts was measured by the spectrophotometric method using Nanodrop spectrophotometer (ND-1000). For EMSA, 10 μg of nuclear proteins from each sample were incubate with ^32^P- radio labeled oligonucleotide probe containing consensus sequence 5'-CGCTTGATGACTCAGCCGGAA-3' that binds AP1 proteins. The DNA-protein complexes were then resolved on 4.5% non-denaturing PAGE, dried and exposed overnight to Kodak X-Omat Films (Kodak India Ltd., India). The quantitative densitometric analysis was performed using Alpha Ease FC version 4.1.0 (Alpha Innotech Corporation, IL). Binding specificity of AP1 probe was checked by using nuclear proteins of HeLa cells with unlabelled 100x molar excess of cold specific competitor (AP1) and non-specific competitor (Oct-1; 5'-TGTCGAATGCAAATCACTAGAA- 3').

### Isolation of whole cell protein and immunoblotting

Whole-cell proteins were isolated as described earlier [28] and protein concentration was determined using Nanodrop spectrophotometer (ND-1000). For immunoblotting, 50 μg/ml of cellular protein/lane was separated on 10% SDS-PAGE and electrotransferred to Immobilin-P membranes (Millipore Corporation, Bedford, MA). Blots were blocked with 5% non-fat milk in PBS containing 0.05% Tween (PBST), rinsed in PBST and incubated overnight at 4°C with the indicated primary antibodies in PBST containing 5% milk. Blots were again washed in PBST and further incubated with HRP-conjugated secondary antibody (Santa Cruz Biotech USA). The bands were visualized using the Luminol reagent detection kit (Santa Cruz Biotech, USA) and exposed to autoradiography film (Kodak). The western blot membranes were re- probed for β- actin expression as an internal control. The quantitative densitometric analysis of the bands was performed using Alpha Ease FC version 4.1.0 (Alpha Innotech Corporation, IL). The expression level of proteins was quantitated with respect to β-actin.

### RNA isolation and northern blotting

The total cellular RNA was extracted from treated and control HeLa cells using TRI reagent (Sigma- Aldrich, USA) according to the manufacturer's instruction. The quality of RNA was estimated by electrophoresis using 2 μl of RNA solution on an ethidium bromide-stained 1% agarose gel in 3-[N- morpholino] propane-sulfonic acid (MOPS) buffer. Concentration of RNA was estimated spectrophotometrically by Nanodrop (NanoDrop Tech, USA). The probes were labeled by the random-priming method using random primer labelling kit (Genei, Bangalore, India) and northern blotting was carried out. Briefly, approximately 15 μg of RNA was resolved on 1% agarose- MOPS formaldehyde gel. Capillary blotted Nylon membrane (IMMOBILON-NY+, Millipore, Bedford, MA) was then UV crosslinked (Hoefer UVC 500 ultraviolet crosslinker, Amersham Biosciences, England) and washed in 6X SSC, air dried, and finally exposed in phosphorimager (Fujifilm FLA-5100, Japan) after pre-hybridization and hybridization in Perfect HYB-PLUS (Sigma Inc, USA) solution as suggested by manufacturer's protocol.

## Results

### Column chromatographic separation, thin layer chromatography and phytochemical analysis of *B. pinnata *leaf extract

Chromatographic separation of *B. pinnata *leaf extracts was performed that produced a total of 6 fractions (Figure 1). Sample from each fraction was spotted on precoated silica gel thin layer chromatographic plate and eluted with chloroform: methanol (95:5) solvent system. TLC plates were observed under visible and UV light. Results showed presence of UV active spots in crude extract, and fraction F1, F3 and F4 (data not shown). The spots with UV activity were found with very low Rf value. The phytochemical analysis of chromatographically separated fractions by Raymond's test for cardiac glycosides and Dragendorff's test for alkaloids were performed that showed presence of alkaloids and steroidal glycosides only in fraction F3 and F4 (Table 1). The F4 was observed to be rich in steroidal glycosides, alkaloids and steroids and F3 gave only a faint coloration in both the reactions. All the other fractions failed to show presence of any of these bioactive compounds.

**Table 1:** Analysis of various chromatographic fraction of *B.pinnata *leaf extract for presence of bioactive phytochemicals

**S. N**.	Test				Colorimetric/turbidity test		
		
		F1	F2	F3	F4	F5	F6
1	Raymond test (Cardiac Glycosides)	-	-	+	++	-	-

2	Dragendorff's test (Alkaloids)	-	-	+	++	-	-

+ - Normal coloration; ++ - Dense coloration, -No change, F1 to F6 designated chromatographic fraction of *B. pinnata *leaf extract.

### Comparative analysis of *B. pinnata *and its fractions for growth inhibitory activity against cervical cancer cells

**Figure 2:**
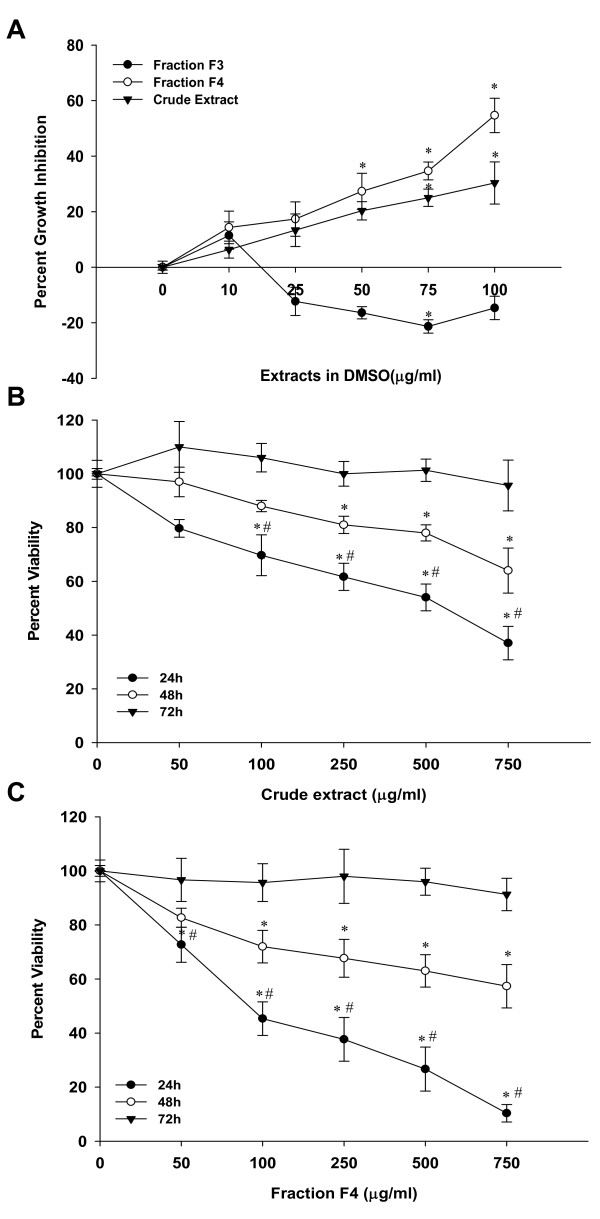
**Dose-dependent effect of *B. pinnata *leaf extract and its chromatographic fraction on the viability of human cervical cancer cells**. **A**. HeLa cells incubated in 96 well plate without or with indicated concentration of *B. pinnata *crude leaf extract or its fraction F3 and F4 were examined for cell viability at 24 h by MTT assay as described in Methods. Percent growth inhibition was measured w.r.t. untreated controls. **B and C**. HeLa cells incubated with increasing concentration of crude extract (B) and fraction F4 (C) were examined for cell viability at 24 h, 48 h and 72 h and their percent viability was measured by MTT assay. The results are representative of three independent experiments with similar results. Values are mean ± SD of the triplicate cultures. *p < 0.05 versus untreated cultures. #p < 0.05 versus cells treated at similar dose of crude extract or fraction F4 treatment at 48 h or 72 h cultures.

*B. pinnata *crude leaf extract and all its chromatographic fractions were examined for cell-growth inhibitory properties on HeLa cells. Cells treated with indicated concentration of test sample were examined by MTT assay for the cell viability. Results shown in Figure 2A demonstrate presence of growth inhibitory property in crude extract and its fraction F4 whereas fraction F3 had minor growth promoting activity. Crude *B. pinnata *leaf extract inhibited cervical cancer cell growth by 30% whereas fraction F4 was comparatively more potent and inhibited cell growth by 55% at 100 μg/ml concentration (Figure 2A). On the other hand, growth of cells treated with fraction F3 was found to be relatively higher compared to control which was maximum (-21.3%) at 75 μg/ml. Further increase in F3 concentration did not increase the growth of cervical cancer cells. Cells treated with other fractions did not show any significant change in their growth properties (data not shown). Based on these preliminary observations, cells were treated with increasing concentration of crude extracts and fraction F4 for 24 h, 48 h and 72 h to examine the dose kinetics and time course of growth inhibition. As shown in Figure 2B and 2C, both the crude extract and fraction F4 showed highest dose-dependent inhibition at 24 h. A longer incubation of treated cultures resulted in partial recovery of growth inhibition at 48 h and complete recovery by 72 h. The interpolated IC50 of crude extract was 552 μg/ml and IC50 of fraction F4 was 91 μg/ml in 24 h treated cultures.

### *B. pinnata *suppress constitutive activation of AP1 in cervical cancer cells

**Figure 3:**
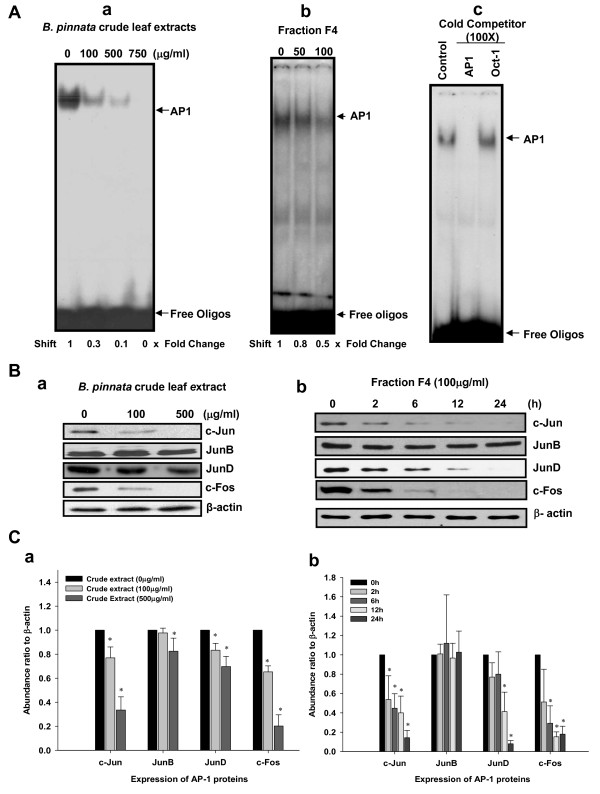
**Effect of *B. pinnata *crude leaf extract on AP1 specific DNA-binding and expression of its constituent subunits in cervical cancer cells**. **A**. Nuclear proteins (10 μg/lane) of HeLa cells treated with indicated concentrations of *B. pinnata *crude extracts **(a) **or its fraction F4 **(b) **for 24 h and were assayed for AP1 DNA binding activity by EMSA. Untreated HeLa nuclear proteins and labeled AP1 probe were incubated in the presence of 100 fold molar excess of unlabeled homologous AP1 probe or heterologous Oct-1 probe and assayed for AP1 specific binding by EMSA **(c)**. Fold change was calculated following densitometric evaluation of shifted band. **B**. Effect of *B. pinnata *leaf extracts on the expression of AP1 family proteins. Representative immunoblots of cellular proteins isolated from HeLa cells treated with *B. pinnata *crude leaf extract **(a) **and fraction F4 **(b) **as indicated showing expression of different members of AP1 family. β-actin served as a loading control. **C**. Aggregated mean (± S.D.) abundance ratios of indicated AP1 proteins w.r.t. to β-actin following treatment with indicated doses of crude extract and fraction F4 in three independent experiments. Band intensity of AP1 proteins and β-actin was measured by densitometry as described in "Methods" and their ratio in untreated control was used as reference. *p < 0.05 versus untreated control cultures.

To assess anti-HPV activity of *B. pinnata *extracts, we next examined the effect of crude extracts and fraction F4 on activity and expression of transcription factor AP1. Nuclear proteins isolated from HeLa cells treated with increasing concentration of *B. pinnata *crude extract and fraction F4 were subjected to EMSA to assess AP1's DNA binding activity. Result in Figure 3A show high levels of constitutively active AP1 in untreated control cells which decreased dose-dependently in cells treated with both *B. pinnata *crude extracts and fraction F4. However, the crude extracts were found to be more effective in abrogating the constitutive activity of AP1 (Figure 3Aa and 3Ab). The specificity of AP1 binding was found to be specific as nuclear extracts of untreated HeLa cells co-incubated with cold competitor, unlabelled AP1 probe, completely quenched the AP1 specific binding to the labeled probe (Figure 3Ac), whereas it remained unaffected in the presence of a heterologous Oct-1 probe. Since loss of AP1 activity could be a direct result of loss of expression of AP1 family protein that forms functional homo/hetero-dimer of active AP1 complex, we checked the effect of *B*. *pinnata *crude leaf extract and fraction F4 on expression of AP1 family proteins, c-Jun, JunB, JunD and c- fos in the treated cells by immunoblotting. Results shown in Figure 3B demonstrate a specific inhibitory effect of crude extracts (3Ba) and fraction F4 (3Bb) on c-Jun and c-Fos whereas treatment of HeLa cells either by crude extract or fraction F4 did not alter expression of JunB. Interestingly, fraction F4-treated cells showed specific inhibition of JunD which was not significantly altered in cells treated with crude extracts.

### Anti-HPV and pro-apoptotic activity of *B. pinnata *fraction F4 in cervical cancer cells

**Figure 4:**
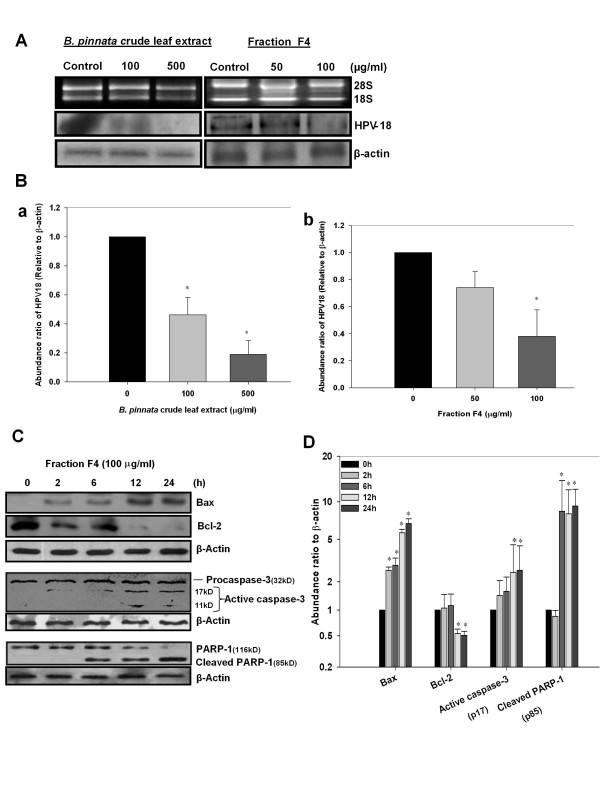
**Anti-HPV and anti-cancer effect of *B. pinnata *crude leaf extract and fraction F4 in cervical cancer cells**. **A**. Northern blot analysis of HPV18 positive HeLa cells incubated with indicated concentrations of crude leaf extract and fraction F4. Quantity and quality of total RNA (15 μg/lane) extracted was examined on agarose gel by assessing 28S & 18S ribosomal RNAs (upper panels). The membranes were hybridized with HPV18-specific probe (middle panel), stripped and rehybridized with β-actin -specific DNA probe as an internal control to assess equal loading (lower panels). **B**. Aggregated mean (± S.D.) abundance ratio of HPV18 transcript w.r.t. to β-actin following treatment with indicated doses of crude extract **(a) **and fraction F4 **(b) **in three independent experiments. The abundance ratio HPV18 transcript to β-actin mRNA was analyzed by densitometry as described in "Methods" and their ratio in untreated control was used as reference. **C**. Immunoblotting analysis of Bcl-2, and Bax, caspase-3, poly (ADP-ribose) polymerase-1 (PARP- 1), in fraction F4 treated HeLa cells at different time-intervals. **D**. The abundance ratio of indicated proteins to β-actin was analyzed by densitometry and their ratio in untreated control was used as reference. The values indicate aggregated mean ± S.D. of three independent experiments. *p < 0.05 versus untreated control cultures.

As crude extract and fraction F4 inhibited pivotal host transcription factor AP1 in cervical cancer cells, in the next part of the investigation, we examined effect of *B. pinnata *crude extract and fraction F4 on transcription of viral mRNA transcripts by Northern blotting using HPV18-specific, radiolabelled probe. Results shown in Figure 4A depict a dose-dependent decrease in the level of HPV18 transcripts in cells treated with crude extracts and fraction F4. Interestingly, despite having lesser cytotoxic activity crude extract was found to be more potent in suppressing HPV18 expression as compared to fraction F4 (Figure 4Ba and 4Bb). As cell toxicity could be outcome of either apoptotic or necrotic death that represents specific phytochemicals-induced programmed cell death or non-specific cell killing respectively, we examined the cells treated with *B. pinnata *fraction F4 for expression of apoptosis-associated proteins like Bax, Bcl-2, Caspase-3 and PARP-1 by immunoblot analysis. Results in Figure 4C demonstrate appearance of fraction F4- induced a gradual increase in expression of proapoptotic Bax and elimination of anti-apoptotic protein Bcl-2 with in 12 h. The changes in Bcl-2 and Bax expression were accompanied by cleavage of procaspase-3 and PARP-1 that represent hallmark features of cells undergoing apoptotic cell death. Caspase-3 activation and cleavage of PARP-1 were found to be late event as they appeared by 24 h and proceeded the alteration in Bax/Bcl-2 levels (Figure 4D).

### Nuclear magnetic resonance and High performance thin layer chromatographic analysis of fraction F4

**Figure 5:**
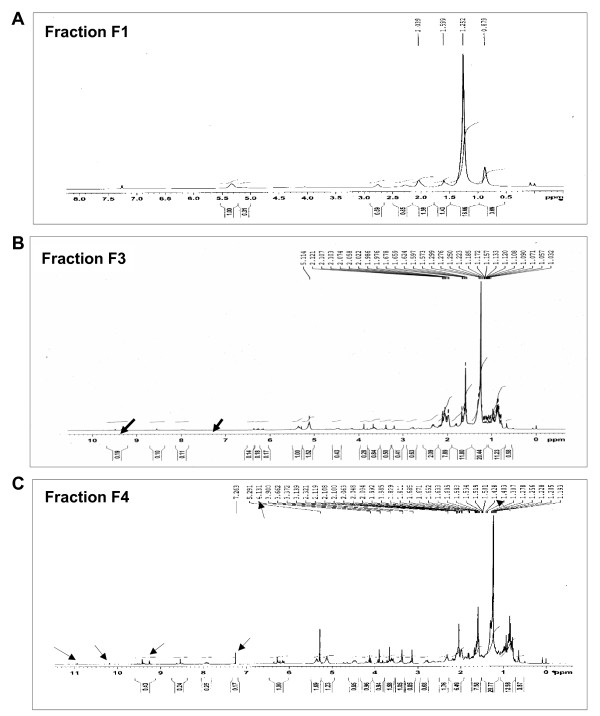
**Functional group analysis of UV active *B. pinnata *fractions by Proton NMR**. Fraction F1, F3 and F4 were dissolved in CDCl3 and NMR was taken at 400 MHz.

**Figure 6:**
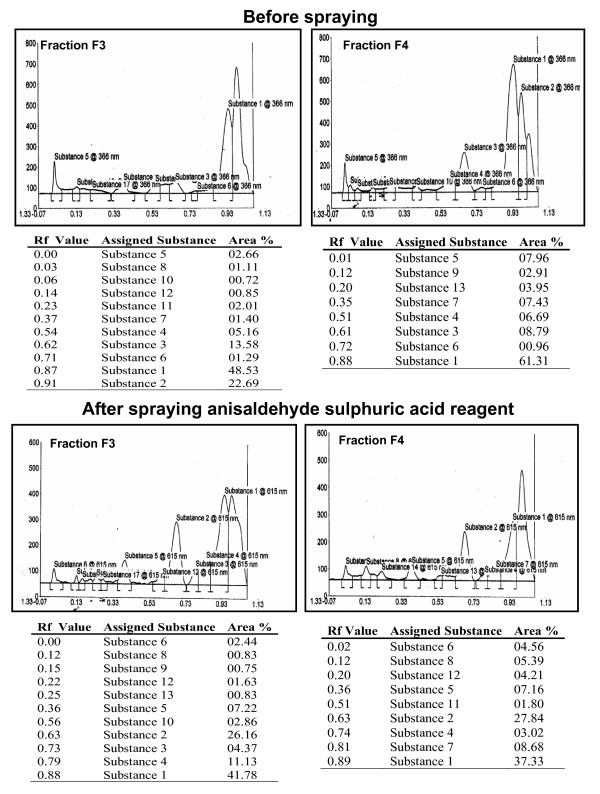
**HPTLC profiling of *B. pinnata *leaf extract, fraction F3 and F4 (A) Finger printing of *B. pinnata *leaf extract, fraction F3 and F4 before and after spraying anisaldehyde sulphuric acid reagent at UV (366 nm) and visible (615 nm) light**. Tables represent Rf value of the detectable substances in fractions with their relative content. Constituent substances were designated with numbers accordingly to their decreasing order of relative abundance.

In an effort to characterize the active principle, fraction F4 was examined by NMR spectroscopy and compared with other UV active fractions, F1 and F3 that served as controls. As shown in Figure 5, NMR spectra of *B. pinnata *leaf extract, fraction F4 was rich in magnitude and number of functional groups present on constituent chemical entities. The NMR spectra of fraction F4 was matched with the reported data which showed presence of specific peaks of functional groups like δ 5.13, 4.36, 1.42 (orthoacetate), δ 10.31 (an aldehyde), δ 7.22, and 6.27 (α-pyrone), δ 4.21 (secondary hydroxyl) but at very low intensity indicating a possibility of presence of low concentration of bryophyllin A. Fraction F4 was further subjected to HPTLC analysis to determine relative ratio of different compounds within this fraction and compared it with fraction F3 to classify the compounds that are unique to F4 only. To analyze and detect compounds with UV activity and terpenoidal structure the HPTLC chromatograms of these fractions were analyzed in two modes, one, without spraying any reagent and visualized at 366 nm wavelength light and second, spraying anisaldehyde sulphuric (AS) reagent and visualized at 615 nm wavelength light. The HPTLC profiles shown in Figure 6 demonstrate presence of 11 substances before and after spraying AS reagent in fraction F3, and 8 & 9 substances in fraction F4 before and after spraying AS reagent, respectively. Constituent substances were designated with numbers accordingly to their order of relative abundance. As summarized in Table 2 before spraying AS reagent there were 5 (substance # 2, R_f _0.91; substance # 8, R_f _0.03; substance # 18, R_f _0.06; substance # 11, R_f _0.23; & substance # 12, R_f _0.14) and 2 (substance # 9, R_f _0.12 & substance # 13, R_f _0.20) substances that were detectable were uniquely present in fraction F3 and F4 respectively that visualized at 366 nm. Whereas after spraying the AS reagent, 4 (substance # 2, R_f _0.63; substance # 9, R_f _0.15; substance # 10, R_f _0.56; & substance # 13, R_f _0.25) and 2 (substance # 7, R_f _0.81; & substance # 11, R_f _0.51) substances were uniquely present in fraction F3 and F4 respectively when visualized at 615 nm.

**Table 2:** Analysis for presence of unique substances in fraction F3 and F4 before and after spraying anisaldehyde sulphuric acid reagent

	*Before Spraying*				*After Spraying Anisaldehyde Sulphuric Acid Reagent*			
**S.N**	**Substance**	**Rf value**	**F3**	**F4**	**Substance**	**Rf value**	**F3**	**F4**

**1**	Substance 2	0.91	+	-	Substance 2	0.63	+	-

**2**	Substance 8	0.03	+	-	Substance 7	0.81	-	+

**3**	Substance 9	0.12	-	+	Substance 9	0.15	+	-

**4**	Substance 10	0.06	+	-	Substance 10	0.56	+	-

**5**	Substance 11	0.23	+	-	Substance 11	0.51	-	+

**6**	Substance 12	0.14	+	-	Substance 13	0.25	+	-

**7**	Substance 13	0.20	-	+	-		-	-

## Discussion

In the present study, we extracted, purified and partially characterized an anti-cancer and anti-HPV molecule/compound from leaves of *B. pinnata *of Indian origin. Using human cervical cancer cells *in vitro *examination of *B. pinnata *crude extracts and its specific chromatographic fractions revealed presence of a growth inhibitory activity that resolved to fraction F4. Further analysis of molecular events revealed a dose-dependent inhibition of constitutively active AP1 by abrogating its DNA-binding activity and expression of AP1 family proteins specifically c-Jun and c-Fos, two components of canonical functional forms of AP1. *B. pinnata *leaf extract suppressed viral transcription of HPV18 in these cervical cells in conjunction with inhibition of AP1 and caused apoptotic cell death as revealed by appearance of Bax, loss of Bcl-2 and cleavage of procaspase-3 to active caspase-3 and proteolytic loss of its target PARP-1. NMR and HPTLC analysis of fraction F4 indicated presence of alkaloids and steroidal glycosides one of which could be bryophyllin A. The chloroform-based extraction procedure used in the present study has been routinely followed by several other studies exploring therapeutic phytochemical [31]. The procedure focused primarily on the chloroform-extracted fraction only and the activity, if any, in the aqueous phase was intentially left out as the same could be potentially contaminating for cell culture as they are likely to possess bacteria and fungal spores and hence could have interfered with the assay. However earlier studies show presence of cytotoxic activities such as bryophyllin B that converges in aqueous phase [31] and could prove useful. Therefore, more refined extraction method are needed to resolve the anti-cancer activity by examining the extract without separation with chloroform as demonstrated by some of the investigators [32] and will be considered in future studies.

A dose-dependent cytotoxic activity observed in leaf extract and its fraction F4 demonstrates a potential therapeutic utility of this medicinal plant against cervical cancer. Earlier studies by Yamagishi et al. 1989 demonstrated presence of similar cytotoxic activity against KB cells in methanolic extracts of *B. pinnata *[31]. Additionally, antitumor activity of *B. pinnata *leaves owing to its antimutagenic activity has also been demonstrated [32]. Interestingly, comparative analysis of fractionated crude leaf extracts in our experiments revealed cytotoxic activity that specifically resolved in pet ether: eth acet::50:50. Earlier studies also show that antimutagenic activity can be extracted in pet ether and ethyl acetate [32]. However, the IC_50 _value derived from HPLC purified active principles from chloroform extracted *B. pinnata *plant in earlier studies [31] were much lower (ranged between 10ηg/ml-4 μg/ml) compared to the one isolated in current study (91 μg/ml). This may be partly due to incomplete separation, but the variability of sensitivity of the cell line used in present study, could also be a major contributor. As a general notion, HeLa cells are much more resistant than many cell lines. Therefore, further studies are warranted on a panel of cell lines to verify the anti-cancer potential of fraction F4. Moreover, the inhibitory effect of both crude extract and fraction F4 were maximal at 24 h but declined thereafter. These observations suggest that active principle might get metabolized or get inactivated during culture process and is no more available to impose growth inhibitory effect. However, these assumptions need further investigation.

In contrast to fraction F4, fraction F3 showed a minor growth promoting activity in dose range 50-100 μg/ml. These results, suggest accumulation of cell growth promoters in F3 fraction that may have coexisted in the plant leaves along with cytotoxic activity. However, there is no report which specifically describes any tumor growth promoting activity associated with this plant.

Our results indicated that *B. pinnata *leaf extracts and its fraction F4 specifically inhibited URR-directed gene expression of HPV18. Constitutively active AP1 by itself has oncogenic outcome because of its regulatory role in several cellular processes like inflammation [33]. Moreover, AP1 is also essentially required for the maintenance of HPV expression through cis-acting binding sites on URR of HPV18. In contrast to higher cytotoxicity potential revealed by fraction F4, the EMSA performed in the present study for assessment of AP1 DNA- binding activity revealed a stronger inhibition of AP1 activity by crude extracts as compared to more cytotoxic fraction F4. Similarly, the inhibition of viral transcription of HPV18 was lesser in cells treated with fraction F4 which was considered to have higher concentration of active principle. These results suggest that fraction F4 of *B. pinnata *leaf extracts might possess two distinct activities which apart from inhibiting activity of transcription factors like AP1 that are responsible for expression of viral oncoproteins (E6 & E7) through binding to URR resulting in oncogenic cell division, can independently prevent cell growth of cancer cells by alternate mechanism(s). This assumption get strength from the finding that cells treated with fraction F4, unlike crude extract, had strongly reduced JunD levels along with reduced c-Jun and c-Fos. Such changes in components of AP1 complex may have varied implication in cellular context and the same could have been translated in higher cytotoxic potential of fraction F4 despite affecting HPV18 transcription at lesser level.

Results of present investigation indicated that cells treated with *B. pinnata *extracts undergo phytochemical-specific programmed cell death/apoptosis and did not induce non-specific necrotic death. A time-dependent appearance of pro-apototic Bax and loss of anti-apoptotic Bcl-2 along with caspase-3 activation and PARP-1 cleavage over 24 h time period strongly supported this assumption. Like, majority of cytotoxic phytochemicals, *B. pinnata *induced apoptotic cell death via intrinsic pathway through loss of mitochondrial membrane potential that are known to be accompanied by Bax expression and loss of Bcl-2 [28,34-36].

In an effort to characterize the active principle through NMR and HPTLC our study revealed that one of the cardiac glycoside that converge in fraction F4 could be bryophyllin-A. However, the study showed presence of high levels of alkaloid that also converge to fraction F4. A number of bioactive molecules including flavanoids, glycosides, steroids, bufadienolides and organic acids have been identified in several studies [10,11,37]. Interestingly, one of the flavanoid glycoside, quercetin analog Quercitrin (quercetin 3- O-alpha- L-rhamnopyranoside) and skapinnatoside, has been shown in several studies to possess anti-leishmanial and anti-inflammatory activities [8,15,38]. In addition, Bufadienolides, bryophyllin-A, -B, -C and bersaldegenin-1, 3, 5-orthoacetate were isolated from the plant of Taiwan origin and was shown to possess hepato-protective activity and cytotoxicity [31,39-41,31]. Apart from this bryophyllol, bryophollone, bryophollenone, bryophynol, 2(9-decenyl) phenenthrene (I), 2(undecenyl) phenenthrene (II), 18α-oleanane, ψ-taraxasterol, α-and β-amyrins and their acetates, 24-epiclerosterol [24(R) stigmasta-5, 25-dien-3β-ol], 24(R) 5α-stigmasta-7, 25-dien-3β-ol, 5α-stigmast-24-en-3β-ol and 25-methyl-5α-ergost-24(28)-en-3β-ol were isolated from the plant leaves and other aerial parts [42,43]. Therefore, it is difficult to conclude from the present study that anti-HPV/anticancer activity observed in crude leaf extract or fraction F4 is displayed specifically by any single chemical entity. Though in unrelated experimental model of EBV-early antigen activation in Raji cells, among 5 different bufadienolides isolated from *kalanchoe pinnata*, showed anti-tumor promoting activity [40] and among them bryophyllin A was found to possess strongest activity. In another study, three new compounds, kalanchosides A-C, as well as five known compounds, were isolated from the aerial parts of *Kalanchoe gracilis*, where all eight isolated compounds showed significant cytotoxic activity against a panel of human tumor cell lines. However, only one of the known compound bryophyllin B inhibited HIV replication in H9 lymphocyte cells [44].

## Conclusion

Our study indicates for the first time that *B. pinnata *can act as an anti-HPV molecule and apoptosis- inducing property. It therefore provides an important lead for development of anti-cancer therapeutics for management of cervical cancer. Further analysis and purification of the *B. pinnata *leaf extract and *in vivo *studies may help in discovering the full potential of *B*. *pinnata *as a source of an effective antiviral/anti-cancer drug.

## Competing interests

The authors declare that they have no competing interests.

## Authors' contributions

S Mahata and S Maru contributed equally to this work. S Mahata and S Maru designed methods and experiments, carried out the laboratory experiments, analyzed the data, interpreted the results and wrote the paper. SS and AP co-worked on western blotting experiments and their interpretation. GM designed and worked on NMR experiments. ACB and BCD defined the research theme, discussed analysis, interpretation, and presentation. All contributing authors seen and approved the manuscript.

## Pre-publication history

The pre-publication history for this paper can be accessed here:

http://www.biomedcentral.com/1472-6882/12/15/prepub

## References

[B1] Tan G Gyllenhaal C Soejarto DD Biodiversity as a source of anticancer drugs Curr Drug Targets 2006 7 3 265 277 10.2174/138945006776054942 16515527

[B2] Akinpelu DA Antimicrobial activity of Bryophyllum pinnatum leaves Fitoterapia 2000 71 2 193 194 10.1016/S0367-326X(99)00135-5 10727819

[B3] Misra SaD SN Antifungal activity of leaf extract of some higher plants Acta Botanica Indica 1979 7 147 150

[B4] Pal S Nag Chaudhuri AK Studies on the anti-ulcer activity of a Bryophyllum pinnatum leaf extract in experimental animals J Ethnopharmacol 1991 33 1-2 97 102 10.1016/0378-8741(91)90168-D 1943181

[B5] Pal S Nag Chaudhuri AK Preliminary studies on the anti-inflammatory and analgesic activities of bryophyllum pinnatum (Lam.) Med Sci Res 1989 17 561 562

[B6] Pal S Nag Chaudhuri AK Further studies on the anti-inflammatory profile of the methanolic fraction of the fresh leaf extract of bryophyllum pinnatum Fitoterapia 1992 63 451 459

[B7] Ojewole JAO Antihypertensive properties of bryophyllum pinnatum (Clam; oken) leaf extracts Am J Hypert 2002 15 4 A34 A39

[B8] Ojewole JA Antinociceptive, anti-inflammatory and antidiabetic effects of Bryophyllum pinnatum (Crassulaceae) leaf aqueous extract J Ethnopharmacol 2005 99 1 13 19 10.1016/j.jep.2005.01.025 15848014

[B9] Umbuzeiro-Valent G Roubicek DA Haebisch EM Mutagenic and antimutagenic evaluation of the juice of the leaves of Bryophyllum calycinum (Kalanchoe pinnata), a plant with antihistamine activity Environ Mol Mutagen 1999 33 4 325 327 10.1002/(SICI)1098-2280(1999)33:4<325::AID-EM10>3.0.CO;2-E 10398381

[B10] Gaind KN Gupta RL Flavonoid glycosides from Kalanchoe pinnata Planta Med 1971 20 4 368 373 5137756 10.1055/s-0028-1099718

[B11] Marriage PB Wilson DG Analysis of the organic acids of Bryophyllum calycinum Can J Biochem 1971 49 3 282 296 10.1139/o71-041 5549729

[B12] Muzitano MF Falcao CA Cruz EA Bergonzi MC Bilia AR Vincieri FF Rossi- Bergmann B Costa SS Oral metabolism and efficacy of Kalanchoe pinnata flavonoids in a murine model of cutaneous leishmaniasis Planta Med 2009 75 4 307 311 10.1055/s-0028-1088382 19085683

[B13] Muzitano MF Tinoco LW Guette C Kaiser CR Rossi-Bergmann B Costa SS The antileishmanial activity assessment of unusual flavonoids from Kalanchoe pinnata Phytochemistry 2006 67 18 2071 2077 10.1016/j.phytochem.2006.06.027 16930642

[B14] Torres-Santos EC Da Silva SA Costa SS Santos AP Almeida AP Rossi-Bergmann B Toxicological analysis and effectiveness of oral Kalanchoe pinnata on a human case of cutaneous leishmaniasis Phytother Res 2003 17 7 801 803 10.1002/ptr.1242 12916081

[B15] Muzitano MF Cruz EA de Almeida AP Da Silva SA Kaiser CR Guette C Rossi-Bergmann B Costa SS Quercitrin: an antileishmanial flavonoid glycoside from Kalanchoe pinnata Planta Med 2006 72 1 81 83 10.1055/s-2005-873183 16450304

[B16] Da-Silva SA Costa SS Rossi-Bergmann B The anti-leishmanial effect of Kalanchoe is mediated by nitric oxide intermediates Parasitology 1999 118 Pt 6 575 582 10406036 10.1017/s0031182099004357

[B17] Cao H Xia J Xu D Lu B Chen G The separation and identification of the flavonoids from the leaves of Bryophyllum pinnatum Zhong Yao Cai 2005 28 11 988 990 16514884

[B18] Parkin DM The global health burden of infection-associated cancers in the year 2002 Int J Cancer 2006 118 12 3030 3044 10.1002/ijc.21731 16404738

[B19] Durst M Gissmann L Ikenberg H zur Hausen H A papillomavirus DNA from a cervical carcinoma and its prevalence in cancer biopsy samples from different geographic regions Proc Natl Acad Sci USA 1983 80 12 3812 3815 10.1073/pnas.80.12.3812 6304740 PMC394142

[B20] Boshart M Gissmann L Ikenberg H Kleinheinz A Scheurlen W zur Hausen H A new type of papillomavirus DNA, its presence in genital cancer biopsies and in cell lines derived from cervical cancer EMBO J 1984 3 5 1151 1157 6329740 10.1002/j.1460-2075.1984.tb01944.x PMC557488

[B21] zur Hausen H Papillomaviruses causing cancer: evasion from host-cell control in early events in carcinogenesis J Natl Cancer Inst 2000 92 9 690 698 10.1093/jnci/92.9.690 10793105

[B22] Chen HC Schiffman M Lin CY Pan MH You SL Chuang LC Hsieh CY Liaw KL Hsing AW Chen CJ Persistence of type-specific human papillomavirus infection and increased long-term risk of cervical cancer J Natl Cancer Inst 2011 103 18 1387 1396 10.1093/jnci/djr283 21900119 PMC3176778

[B23] Zur Hausen H Papillomaviruses and cancer: from basic studies to clinical application Nat Rev Cancer 2002 2 5 342 350 10.1038/nrc798 12044010

[B24] Satoru Kyo DJK Masaki Inoue Taro Kanaya Laimins Laimonis A Expression of AP1 during cellular differentiation determines human papillomavirus E6/E7 expression in stratified epithelial cells J Gen Virol 1997 78 401 411 9018063 10.1099/0022-1317-78-2-401

[B25] Butz K Hoppe-Seyler F Transcriptional control of human papillomavirus (HPV) oncogene expression: composition of the HPV type 18 upstream regulatory region J Virol 1993 67 11 6476 6486 8411351 10.1128/jvi.67.11.6476-6486.1993 PMC238084

[B26] Prusty BK Das BC Constitutive activation of transcription factor AP-1 in cervical cancer and suppression of human papillomavirus (HPV) transcription and AP-1 activity in HeLa cells by curcumin Int J Cancer 2005 113 6 951 960 10.1002/ijc.20668 15514944

[B27] Bharti AC Shukla S Mahata S Hedau S Das BC Anti-human papillomavirus therapeutics: facts & future Indian J Med Res 2009 130 3 296 310 19901439

[B28] Mahata S Bharti AC Shukla S Tyagi A Husain SA Das BC Berberine modulates AP-1 activity to suppress HPV transcription and downstream signaling to induce growth arrest and apoptosis in cervical cancer cells Mol Cancer 2011 10 39 10.1186/1476-4598-10-39 21496227 PMC3098825

[B29] Shukla S Bharti AC Hussain S Mahata S Hedau S Kailash U Kashyap V Bhambhani S Roy M Batra S Elimination of high-risk human papillomavirus type HPV16 infection by 'Praneem' polyherbal tablet in women with early cervical intraepithelial lesions J Cancer Res Clin Oncol 2009 135 12 1701 1709 10.1007/s00432-009-0617-1 19526249 PMC11844788

[B30] Zhengyin Yan GC Zhengyin Yan GWC Evaluation of cytochrome P450 inhibition in human liver microsomes Optimization in drug discovery: in vitro methods 2004 Totowa, NJ: Humana press 231 244

[B31] Yamagishi T Haruna M Yan XZ Chang JJ Lee KH Antitumor agents, 110. Bryophyllin B, a novel potent cytotoxic bufadienolide from Bryophyllum pinnatum J Nat Prod 1989 52 5 1071 1079 10.1021/np50065a025 2607348

[B32] Emmanuel E Obaseiki-Ebor KO Telikepalli Hannumaiah Mitscher Lester A Delbert M Shankel: Antimutagenic activity of extracts of leaves of four common edible vegetable plants in Nigeria (West Africa) Mutat Res Lett 1993 302 2 109 117 10.1016/0165-7992(93)90012-K 7684505

[B33] Eferl R Wagner EF AP-1: a double-edged sword in tumorigenesis Nat Rev Cancer 2003 3 11 859 868 10.1038/nrc1209 14668816

[B34] el SA Arafa Zhu Q Shah ZI Wani G Barakat BM Racoma I El-Mahdy MA Wani AA Thymoquinone up-regulates PTEN expression and induces apoptosis in doxorubicin-resistant human breast cancer cells Mutat Res 2011 706 1-2 28 35 10.1016/j.mrfmmm.2010.10.007 21040738 PMC3037029

[B35] Bharti AC Donato N Singh S Aggarwal BB Curcumin (diferuloylmethane) down-regulates the constitutive activation of nuclear factor-kappa B and IkappaBalpha kinase in human multiple myeloma cells, leading to suppression of proliferation and induction of apoptosis Blood 2003 101 3 1053 1062 10.1182/blood-2002-05-1320 12393461

[B36] Halder B Bhattacharya U Mukhopadhyay S Giri AK Molecular mechanism of black tea polyphenols induced apoptosis in human skin cancer cells: involvement of Bax translocation and mitochondria mediated death cascade Carcinogenesis 2008 29 1 129 138 17984116 10.1093/carcin/bgm233

[B37] El Abdellaoui S Destandau E Toribio A Elfakir C Lafosse M Renimel I Andre P Cancellieri P Landemarre L Bioactive molecules in Kalanchoe pinnata leaves: extraction, purification, and identification Anal Bioanal Chem 2010 398 3 1329 1338 10.1007/s00216-010-4047-3 20714893

[B38] Anjoo Kamboj aAKS Bryophyllum pinnatum (Lam.) Kurz.: Phytochemical and pharmacological profile: A review Pharmacognosy Rev 2009 3 6 364 374

[B39] Yamagishi T Yan XZ Wu RY McPhail DR McPhail AT Lee KH Structure and stereochemistry of bryophyllin-A, a novel potent cytotoxic bufadienolide orthoacetate from Bryophyllum pinnatum Chem Pharm Bull(Tokyo) 1988 36 4 1615 1617 10.1248/cpb.36.1615 3416378

[B40] Supratman U Fujita T Akiyama K Hayashi H New insecticidal bufadienolide, bryophyllin C, from Kalanchoe pinnata Biosci Biotechnol Biochem 2000 64 6 1310 1312 10.1271/bbb.64.1310 10923811

[B41] Supratman U Fujita T Akiyama K Hayashi H Murakami A Sakai H Koshimizu K Ohigashi H Anti-tumor promoting activity of bufadienolides from Kalanchoe pinnata and K. daigremontiana x tubiflora Biosci Biotechnol Biochem 2001 65 4 947 949 10.1271/bbb.65.947 11388478

[B42] Toshihiro Akihisa WCMCK Tamura Toshitake Matsumoto Taro Sterols ofKalanchoe pinnata: First report of the isolation of both C-24 epimers of 24-alkyl-Δ25-sterols from a higher plant Lipids 1991 26 8 660 665 10.1007/BF02536432

[B43] Salimuzzaman Siddiqui SF Siddiqui Bina S Naheed Sultana Triterpenoids and phenanthrenes from leaves of Bryophyllum pinnatum Phytochemistry 1989 28 9 2433 2438 10.1016/S0031-9422(00)97999-8

[B44] Wu PL Hsu YL Wu TS Bastow KF Lee KH Kalanchosides A-C, new cytotoxic bufadienolides from the aerial parts of Kalanchoe gracilis Org Lett 2006 8 23 5207 5210 10.1021/ol061873m 17078679

